# Cocaine

**Published:** 1885-10

**Authors:** 


### COCAINE.

This drug has at last found its proper place in the dental pharmacopœia, and is now considered essential to every well ordered office. We no longer look to it for the accomplishment of miracles, but in the obtunding of dental tissues it plays an important part. Teeth cannot be painlessly extracted by its use, nor does it in every case make the preparation of cavities for filling a perpetual joy. But it will materially alleviate the severity of many painful operations, and no operative dentist is fulfilling his whole duty to his patients unless he has it at hand.

Many of the strictures upon its ineffectiveness have been due to weak or deteriorated preparations. The hydrochlorate very soon degenerates under ordinary circumstances. Some have purchased a four per cent. solution, and after a few days have found it utterly without virtue. More than one such solution had spoiled in our case, but we finally procured from Parke, Davis & Co., the well known manufacturing chemists, small bottles of the hydrochlorate crystals, the hydrobromate crystals, and solutions of the salicylate, five per cent., the citrate, four per cent., and the oleate, five per cent. These have never failed to produce the results that one had a right to anticipate. The oleate has proved the most generally useful, and in many cases has secured unexpected benefits. The salicylate, too, has, in some instances, been effective as an obtundent, when even the oleate had failed.

Perhaps the most pleasing results have been obtained in the removing of living pulps, and in the painless cleaning out of root canals. In one memorable instance, that of a lady, a former patient, who is now living in a distant city, and who had but one day's time for operations, a living, intensely sensitive, inflamed, exposed pulp was found. By the aid of a minute quantity of the muriate crystals, with which a nearly saturated solution was made for immediate application, the pulp was removed absolutely without pain, and the root and tooth were filled at the same sitting, nor has there since been any soreness or irritation.

We can heartily recommend the preparations of Parke, Davis & Co., and we hope that the readers of this journal will obtain some of them, and give them a thorough trial.

